# The Role of NADPH Oxidase in the Inhibition of *Trichophyton rubrum* by 420-nm Intense Pulsed Light

**DOI:** 10.3389/fmicb.2017.02636

**Published:** 2018-01-09

**Authors:** Hao Huang, Weibiao Lv, Ying Chen, Xiufeng Zheng, Yong Hu, Ruihua Wang, Meiling Huang, Hongfeng Tang

**Affiliations:** ^1^Department of Dermatology, Shunde Hospital, Southern Medical University, Foshan, China; ^2^Clinical Laboratory, Shunde Hospital, Southern Medical University, Foshan, China; ^3^Department of Dermatology, Central Hospital of Nanchong, The Second Clinical School of North Sichuan Medical College, Nanchong, China

**Keywords:** 420-nm intense pulsed light, *Trichophyton rubrum*, oxidative stress, NADPH oxidase, keratinase

## Abstract

**Objectives:** To evaluate the effect of intense pulsed light (IPL) on *Trichophyton rubrum* and investigate its mechanism of action.

**Methods:** The viability of fungi treated with IPL alone and with IPL combined with an NADPH oxidase inhibitor (DPI) pretreatment was determined by MTT assays. The reactive oxygen species (ROS) were quantified with a DCFH-DA fluorescent probe. Malondialdehyde (MDA) content and superoxide dismutase (SOD) and glutathione peroxidase (GSH-Px) activities were determined by commercial kits. The transcription of the Nox gene was quantified using quantitative real-time PCR (qRT-PCR) analysis, and micromorphology was observed using scanning electron microscopy (SEM). In addition, fungal keratinase activity was detected by measuring dye release from keratin azure.

**Results:** The growth declined with statistical significance after 6 h of treatment (*P* < 0.001). The ROS and MDA content increased after IPL treatment, whereas the SOD and GSH-Px activity decreased. Nox gene expression was upregulated, and the micromorphology was damaged. Keratinase activity decreased. Fungi that received DPI pretreatment exhibited contrasting outcomes.

**Conclusion:** We found that 420-nm IPL significantly inhibited the growth and pathogenicity of *T. rubrum in vitro*. A suggested mechanism involves Nox as a factor that mediates 420-nm IPL-induced oxidative damage of *T. rubrum.*

## Introduction

*Trichophyton rubrum* is one of the most common dermatophytes and causes a range of superficial fungal diseases that infect keratinized tissues such as skin, hair and nails (Aly, 1994; Burmester et al., 2011; Nenoff et al., 2014). Although dermatophytes can affect healthy humans, they have greater incidence and severity in immunodeficient individuals (Almeida, 2008; Degreef, 2008; Peres et al., 2010). The current management strategies for *T. rubrum* infections include systemic and topical antifungal pharmaceutical treatments (Meis and Verweij, 2001). However, treatment methods that are more effective, convenient and safe should be sought because of several factors such as increasing antimicrobial resistance rates, poor patient compliance because of long-term management, various side effects from the systemic use of antifungal medications and the unsuitability of drug treatment for some patients (such as pregnant women) (Niewerth and Korting, 1999; Schafer-Korting, 2003; Thappa, 2007; Peres et al., 2010; Piscitelli et al., 2011; Wang et al., 2014). Antimicrobial photodynamic therapy (aPDT), currently considered a treatment for infectious disease such as skin diseases and oral diseases (Cieplik et al., 2014a), has been developed in recent years into an effective treatment of *T. rubrum* infections (Baltazar et al., 2013, 2015). Two types of aPDT can occur, type-I and type-II. In type-I reactions, electron transfer between the photosensitizer (PS) and biological molecules results in harmful reactive intermediates such as the superoxide anion (O_2_^⋅-^), hydrogen peroxide (H_2_O_2_) and hydroxyl radicals (⋅OH). In type-II reactions, energy transfer excites the PS to a triplet state, resulting in the production of singlet oxygen, which is an extremely powerful oxidant with a very short lifetime that can react with several biomolecules, such as lipids and proteins (Nyman and Hynninen, 2004; Baltazar et al., 2013).

We also found that light therapy alone also has significant antibacterial effects on microorganisms (Pummer et al., 2017). There are reports suggesting that light from the visible spectrum alone might lead to an autophotosensitization process inducing production of ROS in pathogens as a result of an accumulation of endogenous substances already present within biofilms or tissue that can act as PS (Cieplik et al., 2014b).

Intense pulsed light (IPL), a technology with a wide range of emission wavelengths (410–1200 nm), has been applied in many fields (Babilas et al., 2010). Because IPL devices emit a range of wavelengths with suitable cut-off filters, they can inhibit *Propionibacterium acnes* (*P. acnes*) and reduce the sebum secretion rate according to an autophotosensitization process of intracellular protoporphyrin IX and mechanisms similar to those of antimicrobial photodynamic therapy with PS (Taub, 2007; Yeung et al., 2007; Fan et al., 2013). However, 420-nm IPL treatment of *T. rubrum* infections has not been reported.

Reactive oxygen species (ROS) such as peroxides (H_2_O_2_), the superoxide anion (O_2_^-^) and hydroxyl radical (⋅OH) are generated during cellular metabolism in responses to irritating and/or harmful substances (Turrens, 2003; Ray et al., 2012). However, overwhelming ROS production can cause oxidative stress, which leads to cellular damage and apoptosis (Devasagayam et al., 2004; Ray et al., 2012). Several studies have demonstrated that with or without a photosensitizer, light therapy has certain inhibitory effects on microorganisms and generates an abundance of intracellular ROS (Wi et al., 2012; Baltazar et al., 2013, 2015; Lam et al., 2014). High levels of ROS, induced by light therapy, may cause oxidative damage, which plays a key role in fungus inhibition.

In eukaryotes, nicotinamide adenine dinucleotide phosphate oxidase (NADPH oxidase, Nox) can induce the generation of ROS. Nox enzymes in mammals have been studied comprehensively. However, Nox are also present in fungi and are involved in several physiological metabolic processes and cell differentiation (Bedard et al., 2007; Takemoto et al., 2007). Fungal Noxs have therefore also drawn attention from researchers.

When *T. rubrum* attacks its host, it can secrete keratinase to decompose protein in the skin, nails, etc. (Samdani et al., 1995; Biswas et al., 2001). Keratinase is therefore considered the most important virulence factor of dermatophytes, and its level can be used to indirectly assess the strength of fungal pathogenicity.

In this study, we hypothesized that the antifungal mechanism of photodynamic therapy and the IPL acne treatment mechanism could be employed to inhibit the growth of *T. rubrum* on the basis of its pathogenesis. In this study, we evaluated 420-nm IPL as a method for treating *T. rubrum* infections and investigated the underlying mechanism.

## Materials and Methods

### Preparation of Isolates

We used two strains of *T. rubrum* for experiments. A clinical isolate of *T. rubrum* was provided by the Sun Yat-sen Memorial Hospital, Guangdong, China. Another *T. rubrum* strain, ATCC4438, was obtained from the ATCC (American type culture collection). Sabouraud dextrose agar (SDA) plates were separately inoculated with each strain and incubated for 7 days at 28°C; following the incubation period, spores were collected by brushing the culture surface with 3 ml of phosphate-buffered saline (PBS) using a sterile glass rod. The resulting spore suspension was filtered through a 40-μM filter (Biologix Group Limited). The microconidia were counted using a hemocytometer at fifty thousand microconidia per ml and incubated at 28°C in growth medium consisting of polypeptone (10 g) and glucose (40 g) in water (1000 ml). Colonies of both strains were used for this experiment as follows: (I) Two individual aliquots (10 μl each) of the *T. rubrum* spore suspension were pipetted onto two separate areas of new SDA plates (two colonies per plate) before IPL irradiation, and (II) the spore suspensions were cultured in 96-well flat-bottomed microdilution plates (100 μL per well, 3 wells per group) for IPL irradiation.

### Light Source

**FIGURE 1:**
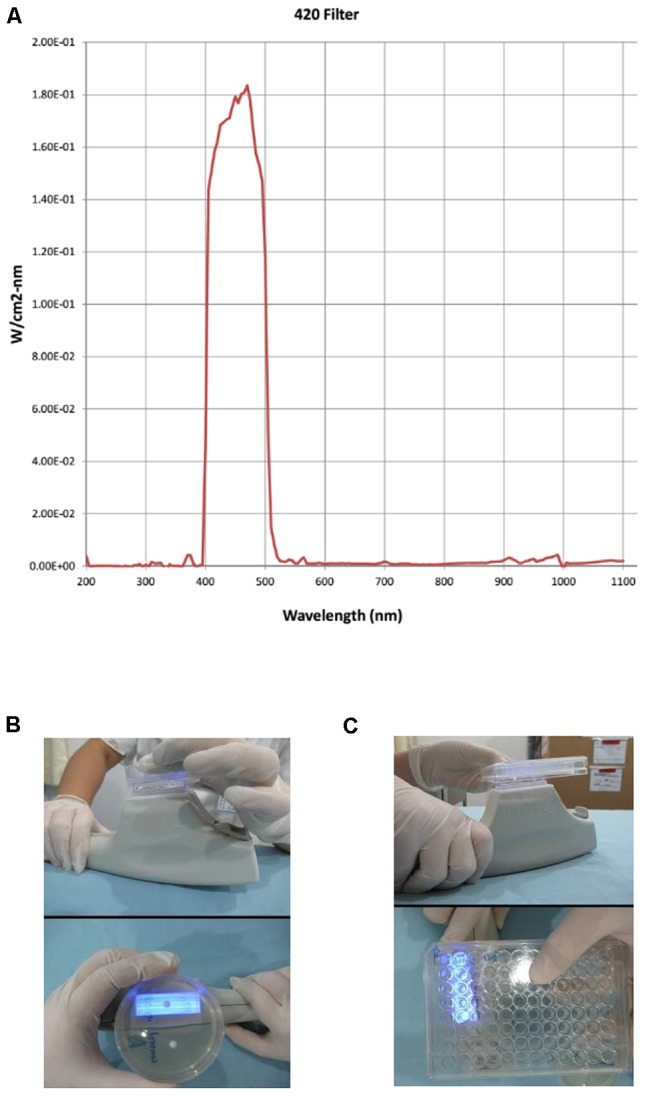
Intense pulsed light (IPL) applicator. The 420 filter emission spectrum of the IPL device **(A)**. Handheld IPL source with a glass applicator during illumination of the whole surface of a colony on agar plates **(B)** and in five wells of a 96-well plate **(C)**.

An IPL device with a xenon lamp light source (Contour Profile, Sciton Company, United States) was used in this study with a cut-off bandpass excitation filter set at 420 nm (**Figure 1**). The setting for each pulse was 150 ms, and the temperature setting was 10°C. Twelve pulses with fluences of 11, 12 and 13 J/cm^2^ were applied to determine optimal conditions.

### Optimization of Conditions for IPL Use on *T. rubrum*

After illumination under the various conditions described above, the fungal viability of both colonial forms was determined using the methods described below. (I) Within 6 h of irradiation, the suspension viability was determined by an MTT assay. MTT reagent (5 mg/mL) was added to each well and then incubated at 28°C for 4 h. After 4 h of incubation, the 96-well plates were centrifuged at 4400 rpm (10°C, Thermo, United States), the supernatant was removed, and 100 μL of DMSO was added. Measurements were collected at 490 nm using a spectrophotometer. (II) The surface areas of both the treated and control colonies on SDA plates determined by photographing them with a digital camera (Canon) before and 3 days after treatment. The areas of the colonies were calculated in terms of pixels using Photoshop CX7 software. The optimal conditions for use of IPL on *T. rubrum* was selected according to the results of the above tests.

### The Effect of Nox on the IPL Treatment of *T. rubrum*

To analyze the role of Nox in IPL treatment, we pretreated the fungus with diphenyleneiodonium chloride (DPI), a Nox inhibitor, for 2 h before IPL treatment and determined fungal viability by MTT assay.

### Determination of ROS Level

Based on the results of the above tests, we divided the cultures grown in liquid medium into a control group, an IPL-treated group (12 pulses at 12 J/cm^2^) and an IPL + DPI group (IPL with 12 pulses at 12 J/cm^2^+ 5 μM DPI). The intracellular total ROS level was evaluated using 10 μM 2′,7′-dichlorofluorescein diacetate (DCFH-DA, Beyotime Biotechnology, Haimen, China), a fluorescent probe. Following IPL treatment, the *T. rubrum* suspension was incubated with DCFH-DA at 28°C for 30 min and then washed twice with PBS. From each sample, 10,000 events were collected using a flow cytometer (BD FACS Canto II). The fluorescent signal intensity was analyzed with an excitation wavelength of 488 nm and monitoring an emission wavelength of 525 nm.

### Determination of MDA Content and SOD and GSH-Px Activities

The malondialdehyde (MDA) content was measured to evaluate the degree of lipid peroxidation. In addition, the activities of intracellular antioxidants superoxide dismutase (SOD) and glutathione peroxidase (GSH-Px) were determined to assess the body’s antioxidant levels. Three tests were used from a commercially available assay kit made by the Nanjing Jiancheng Bioengineering Institute. After IPL treatment, the suspensions of *T. rubrum* were harvested and homogenized in an ultrasonic disintegrator (JY92-IIN, Ningbo Scientz Biotechnology, Ningbo, China) in ice-cold PBS. The suspension was centrifuged to obtain the supernatant used to determine the MDA content by the thiobarbituric acid (TBA) method, the SOD activity by the WST-1 method and the GSH-Px activity by a colorimetric method according to the manufacturer’s instructions. The results of the above assays were combined with protein concentrations determined using a BCA protein assay kit (Beyotime Biotechnology, Haimen, China).

### qRT-PCR Analysis of the Nox Gene in *T. rubrum*

The suspension of *T. rubrum* was sampled and ground in liquid nitrogen. Total RNA was extracted using a HiPure Fungal RNA Kit (Magen, Guangzhou, China) following the manufacturer’s instructions. The extracted RNA was treated using a DNase On Column Kit (Magen, Guangzhou, China) at 37°C for 30 min to remove the genomic DNA. Subsequently, a PrimeScript^TM^ RT Master Mix Kit (Takara, Dalian, China) was used for reverse transcription. β-Tubulin of *T. rubrum* was selected as the reference gene. The gene-specific primers were designed using Primer 5.0 Software. For β-tubulin, the primers were β-tubulin-FP, 5′-CCGCTCTTTGCTCTATTCCT-3′, and β-tubulin-RP, 5′-CCATCTCGTCCATACCCTCA-3′. For NADPH oxidase (Nox), the primers were Nox-FP, 5′-TGGCTGTGACTTTGACGAGA-3′, and Nox-RP, 5′-CCGACTAACACCGCTACTTC-3′. The qRT-PCR experiment was conducted using a SYBR Premix Ex Taq^TM^ Kit (Takara, Dalian, China) and a LightCycler^®^ 480 II system (Roche, Mannheim, Germany). All values were normalized to those of β-tubulin expression. Relative expression was analyzed using the comparative Ct method (2^-ΔΔC_T_^). A probability (*p*) value ≤ 0.05 was considered to indicate significance differences. (Only strain ATCC4438 was used in the qRT-PCR analysis.)

### SEM Examination of *T. rubrum* after IPL Treatment for 24 h

The colonies were cut with a sterile blade, and the pieces of the DPI group were incubated in PBS with DPI for 2 h as described by Xing et al. (Xing et al., 2013). The pieces were treated with IPL, and after 24 h, the samples were washed twice and fixed with 2.5% glutaraldehyde for 48 h at 4°C. The samples were air-dried, sputtered-coated with gold-palladium and observed and photographed under a Hitachi S-3000N Scanning Electron Microscope (Japan).

### Determination of Fungal Keratinase Activity

We added 500 μl of suspension (15000 microconidia per ml) into soy peptone medium at 28°C for 10 days. We divided the cultures grown in liquid medium into a control group, an IPL group (12 pulses at 12 J/cm^2^), an IPL+DPI group (IPL 12 pulses at 12 J/cm^2^+ 5 μM DPI) and a DPI group (5 μM DPI). Keratinase activity was measured using keratin azure (Sigma). Supernatants of samples (3 ml from each group) were incubated with keratin azure (10 mg) at 37°C for 72 h in 2 ml of buffer (0.555 g CaCl2 in 50 ml of pH 8.0 Tris-HCl). Keratinase activity was determined by measuring the absorbance at 595 nm.

### Data Analysis

All data were expressed as the mean ± SD. Statistical analyses were performed using a paired-sample *t*-test and one-way analysis of variance (ANOVA), followed by *post hoc* analysis using the least significant difference (LSD) test or Dunnett’s T3 test. A *P*-value < 0.05 was considered to indicate a statistically significant difference.

## Results

### Establishing the Optimal Conditions for IPL against *T. rubrum*

**FIGURE 2:**
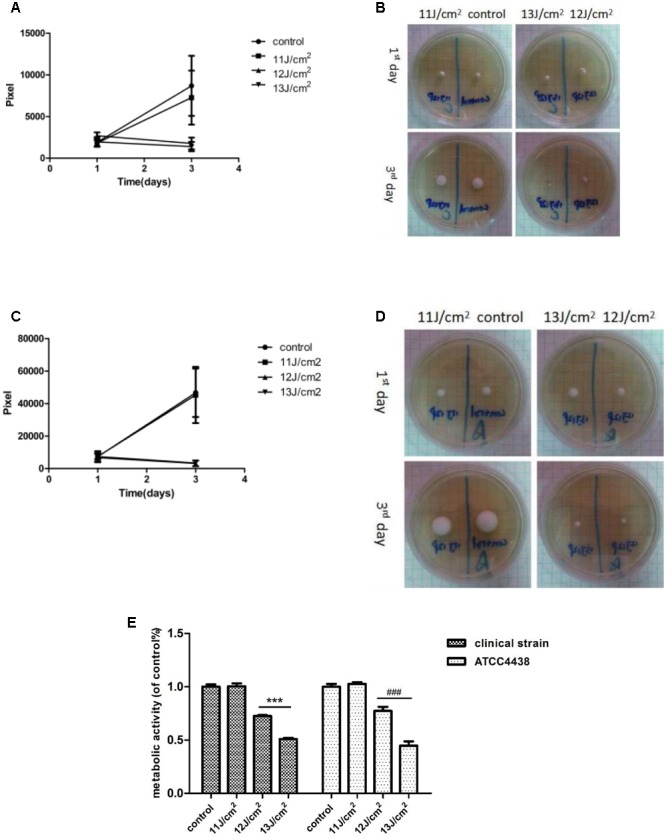
Growth of *T. rubrum* at different light doses of IPL treatment. (**A,B**, clinical strain; **C,D** ATCC4438). **(A)** Is a pixel line chart based on the samples in **(B)** (clinical strain). **(C)** Is a pixel line chart made based on the samples in **(D)** (ATCC4438). **(E)** Fungal viability was evaluated by MTT assays 6 h after IPL treatments. The data are reported as the mean of six independent experiments. ^∗∗∗^*P* < 0.001 vs. untreated clinical strain, ^###^*P* < 0.001 vs. untreated ATCC4438.

Results from a range of IPL doses were evaluated to determine the optimal conditions. Light doses of 12 and 13 J/cm^2^ (12 pulses) significantly decreased the viability of both the clinical and *T. rubrum* ATCC4438 strains (*p* < 0.001), whereas 12 pulses of 11 J/cm^2^ had no inhibitory effects on *T. rubrum* compared to the untreated fungus (*p* > 0.05) as determined by both MTT assays and the areas of the colonies in terms of pixels (**Figures 2A–E**). Based on these results, IPL of 12 pulses at 12 J/cm^2^ was selected for further analyses.

### The Effect of Nox on *T. rubrum* Activity after IPL Treatment

**FIGURE 3:**
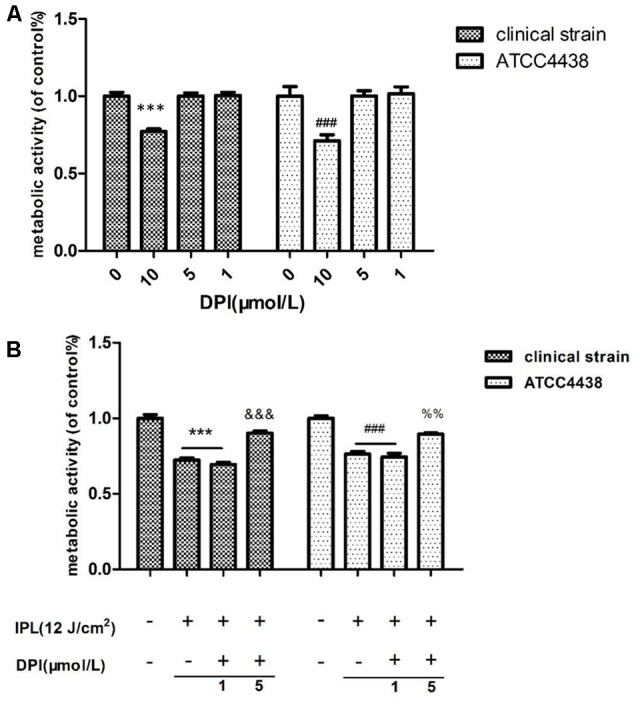
Effect of NADPH oxidase inhibitor on *T. rubrum*. **(A)** Shows the effects of different DPI concentrations alone. **(B)** Shows the effect of DPI on *T. rubrum* activity after IPL treatment. (^∗∗∗^*P* < 0.001 vs. untreated clinical strain, ^&&&^*P* < 0.001 vs. clinical strain IPL group; ^###^*P* < 0.001 vs. untreated ATCC4438; ^%%^*P* < 0.01 vs. ATCC4438 IPL group). The data are reported as the mean of six independent experiments.

Fungal viability was not affected by 1 and 5 μM DPI after the addition or Nox inhibitor at different concentrations (1, 5, and 10 μM), whereas 10 μM DPI decreased viability (**Figure 3A**). Based on these results, experiments with DPI concentrations of 1 and 5 μM were analyzed further.

The Nox inhibitor group was incubated at different concentrations of DPI (1 and 5 μM) with further IPL treatment (12 pulses at 12 J/cm^2^). The MTT assays showed that the fungal viability with inhibitor was significantly higher than that with the IPL treatment alone (*P* < 0.001) (**Figure 3B**). However, samples receiving DPI (1 μM) pretreatment and IPL treatment alone did not statistically significantly differ.

### IPL Induces ROS Generation in *T. rubrum*

**FIGURE 4:**
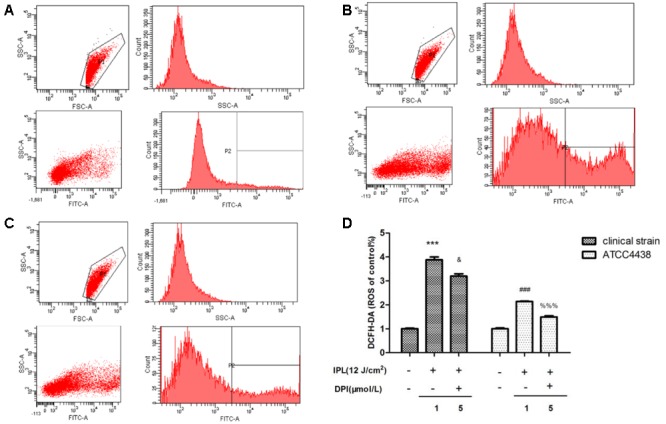
Changes in intracellular ROS levels in *T. rubrum*. **(A)** Is the exemplary dot plots of control group (clinical strain) of flow cytometry. **(B)** Is the exemplary dot plots of IPL group (clinical strain) of flow cytometry. **(C)** Is the exemplary dot plots of IPL + DPI group (clinical strain) of flow cytometry. **(D)** Is the changes of ROS in each groups. (^∗∗∗^*P* < 0.001 vs. untreated clinical strain, ^&^*P* < 0.05 vs. clinical strain IPL group; ^###^*P* < 0.001 vs. untreated ATCC4438, ^%%%^*P* < 0.001 vs. ATCC4438 IPL group). The data are reported as the mean of three independent experiments.

In this study, we measured the levels of ROS by flow cytometry in two *T. rubrum* strains after IPL treatment. The two gates of flow cytometry were set by engineer of BD company (**Figures 4A–C**). P1 is all fungal groups, P2 is a target fungal group (relative fluorescence intensity of expression). The IPL group (12 pulses at 12 J/cm^2^) showed significantly more (*P* < 0.001) fluorescence than the control group (100%). The clinical strain and ATCC4438 produced 388 and 214%, of the control strain’s fluorescence, respectively. However, the IPL + DPI group (IPL 12 pulses at 12 J/cm^2^+ 5 μM DPI) exhibited markedly less ROS generation (320 (clinical strain) and 149% (ATCC4438) of the control group levels) than the IPL group (**Figure 4D**).

### Effect of IPL on MDA Content and SOD and GSH-Px Activities

**FIGURE 5:**
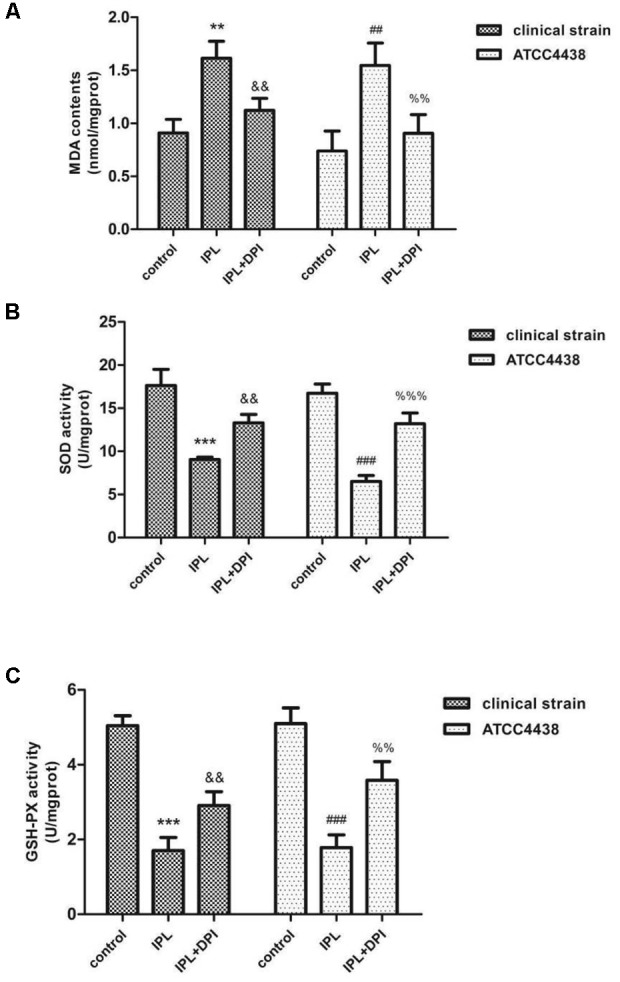
Changes in MDA content **(A)** and SOD **(B)** and GSH-Px activities **(C)** in *T. rubrum*. (^∗∗∗^*P* < 0.001 vs. untreated clinical strain, ^&&^*P* < 0.01 vs. the clinical strain IPL group; ^###^*P* < 0.001 vs. untreated ATCC4438, ^%%^*P* < 0.01 vs. ATCC4438 IPL group, ^%%%^*P* < 0.001 vs. ATCC4438 IPL group). The data are reported as the mean of three independent experiments.

We determined the MDA content and SOD and GSH-Px activities in the two *T. rubrum* strains after IPL treatment. In the clinical strain and ATCC4438, the levels of MDA increased to 177 and 209% of their control values, respectively, after IPL treatment alone (IPL group). However, pretreatment with DPI (IPL + DPI group) decreased the production of MDA to 123% (clinical strain) and 122 (ATCC4438) of that of the controls (**Figure 5A**). The IPL group showed significantly less SOD and GSH-Px activity than the control group, whereas the IPL + DPI group exhibited more of both than the IPL group. The SOD activity of the IPL group was 51 (clinical strain) and 39% (ATCC4438), of the control group values, and the GSH-Px activity was 34 (clinical strain) and 35% (ATCC4438). However, the IPL + DPI group demonstrated markedly higher SOD (75 (clinical strain) and 79% (ATCC4438) of the control values) and GSH-Px activity (58 (clinical strain) and 70% (ATCC4438) of the control values) than the IPL group (**Figures 5B,C**).

### Nox Gene Expression in *T. rubrum*

**FIGURE 6:**
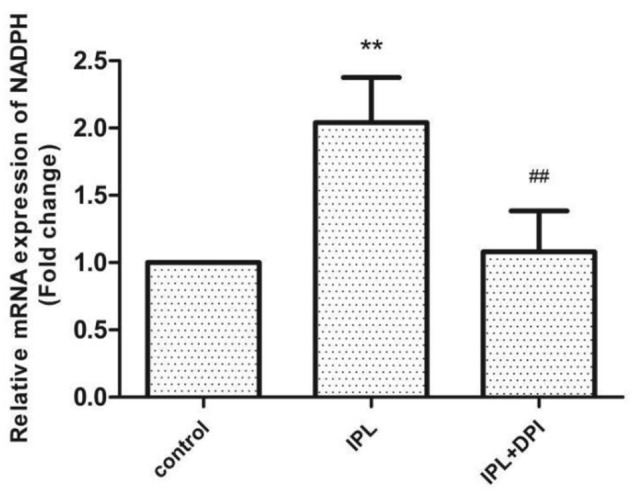
Nox gene expression in *T. rubrum.* (^∗∗^*P* < 0.01 vs. untreated ATCC4438, ^##^*P* < 0.01 vs. ATCC4438 IPL group; mRNA levels are normalized to those of *P*-tubulin). The data are reported as the mean of three independent experiments.

qRT-PCR results indicated that the expression of the Nox gene in the IPL group was significantly upregulated compared with that in the control group (*P* < 0.01), but the level of mRNA was not significantly different in the IPL + DPI and control groups (*P* > 0.05) (**Figure 6**).

### Examining Morphological *T. rubrum* Changes by SEM

**FIGURE 7:**
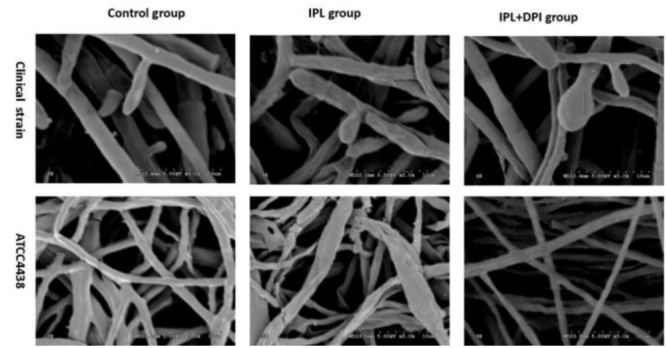
Scanning electron micrographs of *Trichophyton rubrum* with IPL treatment alone and DPI pretreatment + IPL treatment for 24 h.

Under an electron microscope, the *T. rubrum* hyphae of both strains were atrophic, distorted, shrunken and irregular, with the emergence of many damaged and broken hyphae and surface deformation after IPL treatment alone for 24 h. However, fungi receiving a DPI pretreatment were uniformly thick, smooth and plump, with almost no damage (**Figure 7**).

### Changes in Fungal Keratinase Activity

**FIGURE 8:**
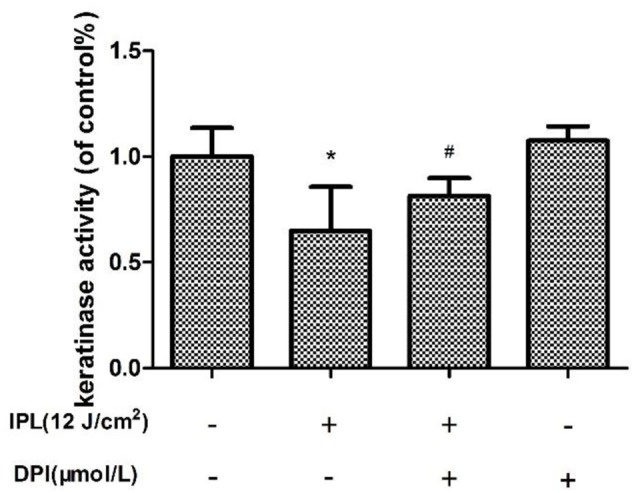
Effect of 24 h of IPL treatment on fungal keratinase activity. (^∗^*P* < 0.05 vs. untreated group, ^#^*P* < 0.05 vs. IPL group). The data are reported as the mean of three independent experiments.

The keratinase activity of samples after IPL treatment alone was less than that of the control group, while it was greater in samples that had received DPI pretreatment. In addition, the difference between the activity of the group with DPI alone and that of the control group was not statistically significant (**Figure 8**).

## Discussion

Several recent reports have described uses of phototherapy on fungal diseases in the clinic. Calzavara-Pinton et al. (2004) reported that 9 patients with refractory toe fungal disease caused by *Candida albicans, T. mentagrophytes*, and *T. rubrum* were treated with ALA-aPDT, and this treatment exhibited good curative effects. Sotiriou et al. (2010) also reported that after ALA-aPDT treatment of 30 cases of onychomycosis, the cure rate was 43.3%. Galvan Garcia HR (Galvan, 2014) found that use of only 1064-nm light from a Nd:YAG Q-switched laser was a safe and effective treatment for onychomycosis, which is mainly caused by *T. rubrum*. LED (370–630 nm) irradiation of *Malassezia* fungus significantly decreased the amount of fungus (Wi et al., 2012). Smijs et al. (2004) observed that after aPDT with a synthesized porphyrin (Sylsens B, DP mme and QDD) was applied to *T. rubrum* conidia, the growth of the fungus was inhibited, and its morphology was damaged. This finding suggests that the use of light therapy alone on superficial skin fungal diseases is beneficial. However, no studies have reported the use of IPL with a 420-nm filter on *T. rubrum* infections. Use of IPL with a specific filter (420 nm) is considered to have anti-inflammatory effects, and several clinical studies have found that IPL at 420 nm has positive effects on acne patients (Kawada et al., 2002; Omi et al., 2004; Lee, 2012). Furthermore, Fan et al. (2013) used 420-nm IPL with light doses as high as 13 J/cm^2^ to treat the skin of rat ears with *Propionibacterium* acne infections *in vitro* in six courses. This treatment not only reduced the number of clinically significant inflammatory lesions but also significantly decreased the expression of tumor necrosis factor alpha (TNF-a) and matrix metalloproteinase 2 (MMP-2); sebaceous glands returned to their normal state in histological examinations.

This study is the first showing that 420-nm IPL inhibits the growth of *T. rubrum*. We found that the growth of both isolates of *T. rubrum* was inhibited after 420-nm IPL treatment. Scanning electron microscopy (SEM) performed on the fungus after IPL irradiation showed that hyphae of both strains were damaged and broken, with surface deformation. However, Ghavam et al. (2015) reported that IPL with 535 and 872 nm light at 20 and 80 J/cm^2^ had little inhibitory effect on the growth of *T. rubrum* colonies. The difference between these outcomes may be related to the use of IPL with different filters differentially inhibiting fungi.

Shapourzadeh et al. (2016) reported that cold atmospheric plasma (CAP) inhibited the growth of *T. rubrum* while moderately increasing keratinase activity, which probably occurred as compensation by the fungus for CAP-mediated fungal growth inhibition. However, we found that IPL inhibited the growth of *T. rubrum* and decreased fungal keratinase activity. The difference between these outcomes may be related to the use of different intervention methods and different detection times. These studies collectively indicate that light therapy may inhibit a variety of fungi for the treatment of dermatomycoses; 420-nm IPL is thus expected to become a new method for superficial fungal disease treatment according to our research.

In this study, we analyzed the mechanism by which IPL shows antifungal effects. This study found that after IPL treatment, the levels of fungal intracellular ROS were consistent with the degree of fungal damage and were more than twofold higher in both strains than the control group. ROS play an important role in cell death in mammals, plants, bacteria, and fungi (Dwyer et al., 2009; Li et al., 2011; Chiappini et al., 2013; Cotoras et al., 2013; You and Chan, 2015). Fungi can produce large amounts of ROS after aPDT or light therapy alone, resulting in fungal damage and death (Wi et al., 2012; Baltazar et al., 2013, 2015). Combined with our study, these results show that ROS play an important role in the antifungal effects of 420-nm IPL against *T. rubrum*.

The biological function of Nox is to transmit electrons and generate ROS (Bedard and Krause, 2007); Nox detects extracellular information through ligand-induced signals, which can stimulate its activation or deactivation to quickly increase or decrease the level of intracellular ROS (Kleniewska et al., 2012). Noxs exist in mammalian cells as well as fungi, plants and other eukaryotes (Bedard et al., 2007). Fungal Nox homologs share sequence similarity with mammalian Noxs (Kim et al., 2011), generate ROS and are suppressed by DPI. Segmüller et al. (2008) and Siegmund et al. (2013) documented that Nox is involved in the sclerotial formation of *Botrytis cinerea*. Xing et al. (2013) found that after the Nox inhibitor DPI was added to *Polyporus umbellatus* (a traditional medicinal mushroom), the production of hydrogen peroxide was lower in hyphae, and the gene expression of NADPH oxidase was downregulated. To investigate whether Nox is involved in the antifungal effects of IPL on *T. rubrum*, we added the inhibitor DPI to a fungal suspension for 2 h of pretreatment before IPL intervention. qRT-PCR showed that Nox gene expression in this group was significantly lower than in fungi with only IPL intervention and similar to the expression level in the control group. The viability of the fungi was significantly higher when they received both DPI pretreatment and IPL treatment than when they received IPL alone. SEM showed that hyphae with DPI pretreatment were almost undamaged. The levels of intracellular ROS were also significantly lower in the IPL + DPI group than the IPL group and reduced antifungal effects on *T. rubrum* from oxidative damage induced by IPL. In addition, the fungus with DPI pretreatment showed higher keratinase activity than members of the IPL group. We speculated that DPI inhibits Nox expression and reduces IPL-induced ROS accumulation to weaken the antifungal effects of IPL and increase keratinase activity. However, the viability and keratinase activity of the IPL + DPI group was lower than those of the control group, whereas the levels of ROS were higher than those of the control group. The increased quantities of ROS and decreased keratinase activity induced by IPL resulted from both the increase in Nox expression and other routes such as mitochondrial-related pathways. However, the upregulation of Nox may regulate ROS increases after IPL treatment, resulting in a relatively lower number of active fungi producing less keratinase activity. DPI may suppress the IPL effect on Nox activation just to reduce the IPL-induced oxidation chain reaction, fungal inhibition and fungal pathogenicity.

Membrane lipid peroxidation that results in a loss of integrity can lead to irreversible cell function damage (Allah et al., 2015). Lipid peroxidation, as measured by the MDA content, is recognized as an important criterion for assessing the magnitude of oxidative stress (Savouré et al., 1999; Andriantsitohaina et al., 2012). The results suggest that the MDA content of the IPL-treated group was significantly greater than that of the control but significantly lower than that of the IPL + DPI group. The enhanced activity of intracellular antioxidants such as SOD and GSH-Px resulting from external stimuli may mediate the rapid removal of ROS and thereby protect cells from possible oxidative damage to maintain the cell oxidation and antioxidant balance (Noctor and Foyer, 1998; Mittler, 2002; Weng et al., 2011). Determining SOD and GSH-Px activities can thus be used to evaluate the ability of eukaryotes to resist oxidative damage. This study shows that 420-nm IPL treatment can decrease SOD and GSH-Px activities in *T. rubrum* and that DPI pretreatment can restore SOD and GSH-Px activity in fungi. This result indicates that Nox inhibitors can effectively inhibit lipid peroxidation and the destruction of antioxidant enzymes caused by 420-nm IPL. We thus speculate that after fungi generated a large number of ROS after 420-nm IPL treatment to cause oxidative stress and increases in MDA content. At the same time, the consumption of antioxidants such as SOD and GSH-Px increased. These results led to injury or even death of *T. rubrum*. However, after the Nox inhibitor DPI had been added, qRT-PCR assays detected the downregulation of Nox gene expression, which resulted in decreased ROS and MDA content and increased SOD and GSH-Px consumption and cell viability. We speculate that Nox is a crucial factor that mediates a response to 420-nm IPL and consequently injures *T. rubrum* by inducing oxidative stress, which can lead to cell damage and death.

In summary, our study showed that 420-nm IPL significantly inhibited the growth of *T. rubrum* and fungal pathogenicity *in vitro*. Although the mechanism of this behavior is not entirely clear, the generation of large amounts of ROS, inducing a cascade of reactions, may play an important role. According to our study we thought that this cascade of reactions is induced by Nox, which may also be one of its targets. And we speculate that the *T. rubrum* may have endogenous porphyrins and occurred autophotosensitization process induced by 420-nm IPL refer to the antimicrobial efficacy of irradiation with visible light on bacteria *in vitro* (Pummer et al., 2017) so that the further exploration is needed. However, based on our findings, 420-nm IPL is a new potential tool to treat superficial fungal diseases caused by *T. rubrum* and possibly those caused by other fungi.

## Author Contributions

HT was responsible for experimental design. HH, WL, YC, XZ, YH, RW, and MH were responsible for experimental operation. HH and HT were responsible for writing article.

## Conflict of Interest Statement

The authors declare that the research was conducted in the absence of any commercial or financial relationships that could be construed as a potential conflict of interest.
